# Experimental Investigation of Tear Strength, Stress Relaxation, and Anisotropy of Polypropylene Surgical Meshes

**DOI:** 10.3390/polym18202595

**Published:** 2026-10-21

**Authors:** Sylwia Łagan

**Affiliations:** Faculty of Mechanical Engineering, PK Krakow University of Technology, 24 Warszawska Street, 31-155 Krakow, Poland; sylwia.lagan@pk.edu.pl

**Keywords:** surgical meshes, tear strength, stress relaxation, anisotropy, viscoelastic behavior, mechanical characterization

## Abstract

In this study, the mechanical behavior of polypropylene surgical meshes was investigated with a focus on tear strength, stress relaxation, and anisotropy. Four commercially available meshes with different structural architectures and grammages were tested. Mechanical characterization was performed by means of trouser tear tests and stress relaxation tests conducted for 1 h at a constant strain of 20%. Tear strength, secant stiffness, apparent Young’s modulus, initial and residual stresses, and normalized relaxation response were determined, and the anisotropic behavior of the meshes was evaluated in two orthogonal directions. Significant differences were observed among the investigated meshes. Materials with higher grammage and more compact structures exhibited greater tear strength, whereas lighter meshes showed lower strength and increased susceptibility to tear propagation. All meshes demonstrated pronounced viscoelastic behavior, characterized by an initial rapid stress decay followed by slower relaxation processes and gradual stabilization. The absolute stress response and stiffness were strongly dependent on mesh structure and loading direction, whereas normalized stress relaxation parameters were largely independent of orientation. The combined evaluation of tear resistance and time-dependent mechanical response highlights the importance of structural parameters and anisotropy in determining the performance of surgical meshes. These findings contribute to a better understanding of mesh mechanical behavior and may support the optimization of mesh design and the development of implants with improved durability and mechanical reliability under physiological loading conditions.

## 1. Introduction

A surgical mesh (mesh implant) is a medical device used to provide additional support to weakened or damaged tissue. It is typically made of a porous structure composed of synthetic or biological materials and designed to reinforce tissue and promote healing. According to the U.S. Food and Drug Administration (FDA), surgical mesh is generally regulated as a Class II medical device, while in the European Union (EU), it is classified as Class IIb or Class III, depending on the intended use [1,2,3]. Surgical meshes can be classified based on their physical and biological properties, including pore size, material origin, architecture, stiffness, and additional functional features [4,5,6].

Surgical meshes are widely used across multiple medical fields, with hernia repair being the most common application, where the mesh reinforces the abdominal wall and reduces the risk of recurrence [5,6,7,8,9,10,11]. Beyond hernia repair, surgical meshes are used for pelvic floor reconstruction, guided bone regeneration, soft tissue repair in complex wounds, and breast reconstruction [11,12,13,14,15]. Mesh implants are manufactured from various materials chosen to provide specific mechanical and biological properties. Polypropylene (PP) is the most widely used non-absorbable polymer because of its favorable mechanical strength, flexibility, and chemical stability [4,5,6,7]. Other commonly used materials are PTFE and PET [5,13], while bioresorbable alternatives include polymers such as PGA and PLA [10,13]. Hydrogel-based composites and biological meshes derived from decellularized tissues represent more advanced solutions, offering improved biocompatibility but often lower mechanical strength [11,14,15,16]. Metallic meshes, particularly titanium meshes, are also used in specific applications such as guided bone regeneration and craniofacial reconstruction, where high structural stability is required [17,18]. To enhance performance, various modification techniques have been developed, such as surface coatings and functionalization strategies that improve biocompatibility and reduce complications including adhesion and infection [15,19,20,21,22]. Recent advances have also resulted in smart meshes and nanofibrous systems designed to better mimic native tissue and respond to environmental stimuli [8,16,23,24].

Despite these developments, the interaction between mesh structure and mechanical performance remains a critical aspect determining clinical outcomes. The properties of surgical meshes depend not only on the material but also on the manufacturing process, which strongly influences their structure and mechanical behavior. Common manufacturing techniques include knitting and weaving [5,7,15], which provide good flexibility and scalability. More advanced approaches, such as electrospinning [8,13] and additive manufacturing, enable the fabrication of complex architectures, although they often exhibit lower mechanical performance [13,25,26,27,28,29].

For clinical effectiveness, meshes must exhibit appropriate mechanical properties. Their behavior under physiological conditions is typically evaluated using mechanical tests such as uniaxial and biaxial tensile testing, which provide information on strength and stiffness, as well as ball burst and suture retention tests, which assess resistance to multidirectional loading and fixation stability [15,30,31,32]. Tear strength is particularly important because it is directly related to the resistance of the mesh to tear propagation and structural failure [15,33].

Although the mechanical properties of surgical meshes, such as stiffness, tensile behavior, and anisotropy, have been extensively investigated, these characteristics are most often described separately and under varying experimental conditions [32,34]. While surgical meshes exhibit viscoelastic properties, including stress relaxation, these time-dependent characteristics remain less extensively studied than conventional mechanical parameters, primarily due to the lack of standardized testing protocols. Studies combining the analysis of tear resistance, stress-relaxation behavior, and directional mechanical anisotropy within a single experimental framework remain limited. The present study provides an integrated assessment of these parameters for commercially available polypropylene meshes with diverse structural architectures. Furthermore, stress-relaxation behavior was characterized using a Prony-series model, enabling comparison of relaxation kinetics among different mesh types and loading directions.

## 2. Materials and Methods

### 2.1. Materials

Four commercially available surgical meshes (Figure 1) from two manufacturers, Yavo Sp. z o.o. (Bełchatów, Poland) and Tricomed Sp. z o.o. (Łódź, Poland), were investigated. All meshes were made of non-absorbable polypropylene and exhibited different grammages and structural architectures. The microstructure was analyzed using a stereoscopic optical microscope (Discovery V8, Zeiss, Oberkochen, Germany) and AxioVision Rel. 4.8 software (Zeiss, Oberkochen, Germany) to characterize fiber orientation, pore geometry, and other structural features. The characteristics of the meshes are summarized in Table 1.

Two types of specimens were prepared: the first, in the form of a strip with dimensions of 10.0 × 60.0 mm, was used for the relaxation test, while the second, in a trouser-shaped configuration, was used for the tear test. Specimens for the tear test were initially cut into strips measuring 40.0 × 90.0 mm and subsequently slit along the longitudinal centerline to half of their length to obtain the trouser tear geometry.

The degree of anisotropy (α) was calculated as the ratio of the parameter measured in the d1 direction to the corresponding value measured in the d2 direction:

(1) $$\alpha =\frac{X_{d1}}{X_{d2}},$$

where X represents the analyzed mechanical parameter (tear strength, stiffness, apparent Young’s modulus, or stress). The anisotropy coefficient was calculated individually for each specimen pair (d1/d2), and the reported value represents the mean ± SD of the obtained ratios. An α value of 1 indicates isotropic behavior, whereas deviations from unity reflect the degree of anisotropy. Because the original mesh sheets differed in geometry and manufacturer-defined orientation, the testing directions were designated as d1 (the direction appearing more susceptible to stretching) and d2 (the direction appearing less susceptible to stretching).

### 2.2. Mechanical Test

All mechanical tests were performed using a Shimadzu AUTOGRAPH AGX-V testing machine (Shimadzu, Kyoto, Japan) equipped with a 1 kN load cell and TRAPEZIUMX-V v.1.4.1 data acquisition software (Shimadzu, Kyoto, Japan). The tear and stress relaxation tests were carried out at room temperature (20 ± 1 °C) and a relative humidity of 40 ± 5%. The specimen width and thickness were measured using an electronic caliper (YT-7201, TOYA S.A. Wrocław, Poland). For calculation purposes, a simplified uniform rectangular cross-section was assumed, neglecting the presence of pores and nodes. Stress (σ, MPa) was calculated as engineering (nominal) stress by dividing the applied force by the initial cross-sectional area of the specimen. Because surgical meshes are highly porous structures, the actual load-bearing area is smaller than the nominal geometric cross-sectional area. Therefore, the calculated stress values should be interpreted as apparent (effective) stresses representing the overall mechanical response of the mesh structure rather than the true stresses within the polypropylene fibers.

To assess the tear resistance and mechanical integrity of mesh implants, trouser tear tests were conducted at a crosshead speed of 100 mm/min and a gauge length of 80 mm. Force (F, N) and crosshead displacement were recorded during the test. Tear strength (TS, N/mm) was determined by dividing the tearing force by the thickness (T, mm) of the surgical mesh. The mean tear force was calculated as the average of the five highest force peaks observed after the initial peak during propagation of the tear [30]. Four specimens were tested for each material and loading direction in the tear tests.

Stress-relaxation tests were conducted over a period of 1 h at a constant tensile strain of 20%. The strain level was selected because abdominal wall tissues may experience deformations approaching 20–25% during physiological trunk movements and because it provides a measurable viscoelastic response while remaining well below the failure strain of the investigated meshes. The test duration of 1 h was chosen to capture both the initial rapid stress decay and the subsequent slower relaxation phase and is consistent with previously reported stress-relaxation protocols for polypropylene surgical meshes [34,37].

The gauge length was 50 mm, and the target strain was reached using a crosshead speed of 50 mm/min. Stress-relaxation data were recorded, and stress-relaxation curves were obtained. Five specimens were tested for each material and loading direction in the stress-relaxation tests. Following the relaxation tests, the apparent Young’s modulus (E, MPa) was determined from the initial loading ramp using stress–strain data up to 10% strain. The modulus was calculated as the slope of the stress–strain curve within this quasi-linear deformation range. Because the calculation was based on the nominal cross-sectional area of the specimen, the resulting values represent an apparent structural modulus of the porous mesh rather than an intrinsic material property of the polypropylene fibers. The secant stiffness (k, N/mm) was determined from the corresponding force–displacement curve during the same loading interval (0–10% strain) as the ratio of the change in force to the corresponding change in displacement (Δ*F*/Δ*u*), using a secant-based approach analogous to that proposed by Maurer et al. [37]. The initial stress ($\sigma_{0}$) and the residual stress ($\sigma_{r}$), corresponding to t=0 s and t=3600 s, respectively, were determined. Both parameters represent apparent stresses calculated using the nominal cross-sectional area of the specimen. The relaxation level (R) was defined as the relative decrease in stress, calculated as:

(2) $$R=\frac{\sigma_{0}-\sigma_{r}}{\sigma_{0}}.$$

Normalized stress-relaxation curves ($\sigma^{nor}$) were used to eliminate the influence of the initial stress level and to enable comparison of the viscoelastic response between materials and directions based on the relative stress decay over time. The normalized stress-relaxation response ($\sigma^{nor}$), equivalent to the relaxation function G(t), was subsequently modeled using the Prony series. In this generalized viscoelastic formulation, the time-dependent stress response is represented as a superposition of exponential decay functions, each associated with a characteristic relaxation time $\tau_{i}$ and a corresponding weighting coefficient $A_{i}$. The normalized relaxation function can be expressed as:

(3) $$G\left(t\right)=\frac{\sigma (t)}{\sigma_{0}}=A_{0}+\sum_{i=1}^{n}A_{i}exp(-\frac{t}{\tau_{i}}),$$

where σ(t) denotes stress at time t, $\sigma_{0}$ is the stress measured immediately after reaching the prescribed strain level (*t* = 0 s), $A_{0}$ represents the elastic (time-independent) component, and $A_{i}$ are the viscoelastic weighting coefficients associated with individual relaxation mechanisms. The parameters $\tau_{i}$ define the characteristic relaxation times associated with different time scales of material response. In the present study, the relaxation data were normalized with respect to the maximum stress value, yielding a dimensionless relaxation function. This normalization removes the influence of absolute stiffness while preserving the intrinsic viscoelastic behavior of the materials. The Prony series parameters were determined by fitting the model to the experimental data using nonlinear regression. To establish the appropriate level of model complexity, both two-term and three-term Prony series models were evaluated. The quality of fit was assessed using the coefficient of determination (COD, $R^{2}$). Although the two-term model provided satisfactory agreement with the experimental data ($R^{2}$ = 0.993–0.996), the three-term model yielded improved agreement ($R^{2}$ = 0.9989–0.9993) and more accurately captured the relaxation behavior over the entire time range, particularly in the intermediate region of the relaxation curves. Consequently, the three-term model was selected for further analysis. The resulting model allows quantification of both the elastic fraction of the response (represented by $A_{0}$) and the time-dependent viscoelastic contributions, enabling comparison of relaxation kinetics between different mesh types. Additionally, the weighted average relaxation time ($\tau_{avg}$) was calculated to provide a single representative parameter describing the overall relaxation kinetics of the mesh. $\tau_{avg}$ was calculated as the amplitude-weighted average of the characteristic relaxation times obtained from the Prony series:

(4) $$\tau_{avg}=\frac{\sum A_{i}\tau_{i}}{\sum A_{i}},$$

where $A_{i}$ are the Prony coefficients, and $\tau_{i}$ are the corresponding characteristic relaxation times. This parameter reflects the average timescale of the viscoelastic relaxation process while accounting for the relative contribution of each relaxation mechanism. All Prony-series fits were performed using normalized relaxation curves. The Prony-series coefficients were determined independently for each specimen and subsequently averaged. Therefore, the sums of the averaged coefficients may not be exactly equal to unity because of parameter averaging and rounding of the reported values.

Data are presented as mean ± standard deviation (SD). Statistical analysis was performed separately for each mesh type to compare the two loading directions (d1 and d2), because the primary objective was to evaluate directional effects rather than to perform a global comparison among all mesh types. Therefore, pairwise comparisons were conducted separately for each material using Welch’s *t*-test, which does not assume equal variances between groups. Statistical significance was established at *p* ≤ 0.05. Specimens were tested in a randomized order to minimize systematic bias. No preconditioning cycles were applied prior to testing.

## 3. Results

### 3.1. Tear Resistance

The load–displacement curves exhibited three characteristic regions: (1) a toe region associated with fiber straightening and reorientation, (2) a quasi-linear elastic region corresponding to mesh stiffness, and (3) a damage region characterized by progressive fiber failure and structural degradation. Representative tear test curves are shown in Figure 2, whereas the calculated tear strength values are summarized in Table 2.

Optomesh Thinlight exhibited the lowest degree of anisotropy among the investigated meshes (α = 0.82 ± 0.09), although a statistically significant directional difference in tear strength was observed. Optomesh Macropore showed comparable tear resistance in both cutting directions (α = 0.94 ± 0.11), indicating quasi-isotropic behavior. In contrast, Yavo meshes demonstrated pronounced directional dependence. Yavo Soft exhibited a tear strength of 39.48 ± 0.79 N/mm in the d1 direction and 29.35 ± 0.55 N/mm in the d2 direction, resulting in an anisotropy coefficient of 1.35 ± 0.02. Similarly, Yavo Normal showed high tear resistance in the d1 direction (51.82 ± 2.25 N/mm) and lower values in the d2 direction (31.66 ± 1.27 N/mm), corresponding to an anisotropy coefficient of 1.67 ± 0.09. For Yavo Normal, the tear response strongly depended on specimen preparation. Specimens in which mesh nodes were not intersected during cutting exhibited a tear strength of 31.66 ± 1.27 N/mm in the d2 direction, whereas specimens with node intersection showed a drastic reduction to 2.08 ± 0.15 N/mm. This finding demonstrates a substantial reduction in tear resistance associated with node intersection.

Maximum displacement values ranged from 83.71 ± 10.61 mm to 133.35 ± 9.48 mm and were accompanied by visible structural changes, including pore elongation and knot tightening. The largest displacement was observed for Yavo Soft in the d1 direction, whereas Optomesh meshes generally exhibited lower deformation levels.

Statistical analysis using Welch’s *t*-test revealed significant directional differences for Optomesh Thinlight (*p* = 0.019), Yavo Soft (*p* < 0.001), and Yavo Normal (*p* = 0.027). No significant directional differences were observed for Optomesh Macropore (*p* = 0.552).

### 3.2. Stiffness and Young’s Modulus

Mechanical parameters, including secant stiffness (k) and apparent Young’s modulus (E), were determined from the slope of the stress–strain curve in the quasi-linear region up to 10% strain. The results are summarized in Table 3.

Optomesh Thinlight exhibited similar stiffness and modulus values in both loading directions, with an anisotropy coefficient close to unity (α = 0.92 ± 0.10), indicating quasi-isotropic behavior. No significant directional differences were observed for either parameter (*p* > 0.05). In contrast, Optomesh Macropore, Yavo Soft, and Yavo Normal displayed significant direction-dependent differences in both stiffness and apparent Young’s modulus (*p* ≤ 0.05). The strongest anisotropic response was observed for Yavo Soft, for which the apparent Young’s modulus increased from 1.75 ± 0.27 MPa in the d1 direction to 13.98 ± 1.47 MPa in the d2 direction. The same mesh also exhibited the lowest stiffness in the d1 direction (0.11 ± 0.02 N/mm) and the lowest anisotropy coefficient (α = 0.11 ± 0.01). Overall, the results demonstrate that stiffness and apparent Young’s modulus are strongly dependent on loading direction for most investigated meshes, whereas Optomesh Thinlight exhibits nearly isotropic mechanical behavior.

### 3.3. Stress Relaxation Behavior

Four characteristic stages were identified: (1) loading ramp, (2) rapid relaxation, (3) slow relaxation, and (4) near-equilibrium response. Such behavior is characteristic of viscoelastic materials subjected to constant strain conditions. Average stress relaxation curves for all tested meshes are presented in Figure 3.

The highest force levels were observed for Yavo meshes loaded in the d2 direction, reaching 16.79 ± 2.69 N for Yavo Soft and 17.89 ± 2.36 N for Yavo Normal (Figure 3c). In contrast, substantially lower force values were recorded in the d1 direction, indicating pronounced anisotropic behavior. Optomesh Thinlight exhibited only minor directional differences, whereas Optomesh Macropore showed a marked increase in load-bearing capacity in the d2 direction. The corresponding stress parameters are summarized in Table 4.

The initial stress ($\sigma_{0}$) ranged from 0.35 to 5.23 MPa, while the residual stress after 1 h of relaxation ($\sigma_{r}$) ranged from 0.13 to 2.14 MPa. The relative stress reduction (R) varied between 0.49 and 0.65, indicating that more than half of the initial stress dissipated during the relaxation process for all investigated meshes.

Statistical analysis using Welch’s *t*-test revealed significant directional differences in both σ_0_ and σ_r_ for Optomesh Macropore (*p* < 0.05), Yavo Soft (*p* < 0.001), and Yavo Normal (*p* < 0.001), whereas no significant differences were observed for Optomesh Thinlight (*p* > 0.05). In contrast, no statistically significant differences in normalized residual stress ($\sigma_{r}^{nor}$) were found between directions for any of the investigated meshes (*p* > 0.05), indicating comparable relative stress-relaxation behavior regardless of cutting orientation.

### 3.4. Prony Series Analysis

Although the relative stress reduction was similar for most mesh types and directions, the relaxation process may still differ in terms of its kinetics. To investigate the time-dependent viscoelastic response in more detail, the normalized relaxation curves were fitted using a Prony series model. This approach enabled quantification of the elastic fraction ($A_{0}$), the relative contributions of individual relaxation mechanisms ($A_{1}$–$A_{3}$), and the corresponding characteristic relaxation times ($\tau_{1}$–$\tau_{3}$). The fitted parameters are summarized in Table 5.

Significant directional differences in the Prony parameters were observed only for $A_{0}$ in Optomesh Thinlight, $A_{1}$ in Optomesh Macropore, and $A_{0}$, $A_{2}$, and $\tau_{1}$ in Yavo Soft (*p* < 0.05). No significant directional differences were found for Yavo Normal.

Three characteristic relaxation times were identified from the Prony series model. For Optomesh meshes, $\tau_{1}$ ranged from 5.9 to 7.7 s, $\tau_{2}$ from 116.9 to 144.5 s, and $\tau_{3}$ from 1232.7 to 1723.1 s. For Yavo meshes, $\tau_{1}$ ranged from 11.5 to 15.9 s, $\tau_{2}$ from 157.0 to 175.4 s, and $\tau_{3}$ from 1373.1 to 1465.6 s. Statistical analysis revealed significant directional differences in $A_{0}$ for Optomesh Thinlight and Yavo Soft, in $A_{1}$ for Optomesh Macropore, and in $A_{2}$ and $\tau_{1}$ for Yavo Soft (*p* < 0.05). No significant differences between directions were observed for Yavo Normal or for the long-term relaxation times $\tau_{2}$ and $\tau_{3}$.

As shown in Figure 4, four characteristic stages were observed during the stress relaxation experiments. Although the relaxation response visually appeared to consist of an initial rapid decay followed by a slower long-term response, the Prony series analysis revealed multiple relaxation processes operating over distinct time scales. The Prony series model accurately reproduced the experimental data over the entire test duration (0–3600 s), confirming its suitability for describing the viscoelastic behavior of the investigated meshes. The calculated weighted average relaxation times ranged from 416 to 568 s, suggesting that the dominant viscoelastic response of the investigated meshes occurs within the first several minutes after loading. Despite differences in structural architecture, the overall relaxation kinetics remained comparable between the investigated materials. Differences between mesh types were primarily associated with the initial relaxation rate and the magnitude of stress reduction, whereas long-term behavior remained relatively similar, as reflected by the comparable $\tau_{2}$ and $\tau_{3}$ values obtained from the model.

## 4. Discussion

The results of this study demonstrate that the mechanical behavior of surgical meshes is strongly governed by their structural architecture, particularly fiber orientation, pore geometry, and node distribution. The observed differences were also influenced by mesh grammage, with meshes of higher areal density generally exhibiting greater tear resistance and load-bearing capacity. However, the effects of grammage could not be considered independently of other structural characteristics, including pore architecture and fiber arrangement. These structural features determine load transfer mechanisms and contribute to the pronounced anisotropy observed in most of the investigated meshes. The present findings further support the growing recognition that mesh performance depends not only on material composition but also on architectural design parameters, including fiber orientation and pore geometry, as emphasized in recent reviews of surgical mesh mechanics and design strategies [4]. Optomesh Thinlight exhibited the lowest degree of anisotropy among the investigated meshes and the most isotropic mechanical response overall, despite a small but statistically significant directional difference in tear strength. In contrast, Optomesh Macropore showed quasi-isotropic tear strength but pronounced directional dependence in stiffness and stress response. In contrast, Yavo meshes demonstrated strong directional dependence. Yavo Normal demonstrated sensitivity to specimen preparation, as node intersection significantly reduced tear strength. These results indicate that local structural discontinuities have a major influence on failure behavior, highlighting the importance of mesh architecture in governing crack initiation and propagation. An additional factor affecting tear resistance was specimen preparation. The results obtained for Yavo Normal showed that intersecting mesh nodes during specimen cutting caused a dramatic reduction in tear strength, from approximately 31.7 N/mm to 2.1 N/mm. This finding indicates that local structural discontinuities introduced during specimen preparation can substantially affect tear initiation and propagation. The observed reduction in tear resistance suggests that mesh nodes are important structural elements contributing to the mechanical integrity of the mesh architecture. Their disruption may alter load transfer mechanisms within the mesh and promote tear propagation.

The anisotropic mechanical behavior observed in this study is consistent with previous reports on warp-knitted meshes, where stiffness and strength vary depending on the loading direction relative to loop orientation [38]. Similar ranges of stiffness and stress values have been reported in the literature [39,40], although direct comparison remains challenging due to differences in mesh structure, testing protocols, and data interpretation methods.

The stiffness values obtained in the present study are consistent with previously reported data for polypropylene surgical meshes. Maurer et al. demonstrated that mesh architecture has a dominant influence on mechanical performance and reported considerable variations in stiffness among commercially available mesh implants [37]. Similar trends were observed in the present study, where substantial differences in stiffness and anisotropy were found despite all investigated meshes being manufactured from polypropylene. Furthermore, the relative stress reduction observed in the present study (49–65%) was comparable to values reported by Kirilova-Doneva et al. [40] for polypropylene-based surgical meshes, confirming the pronounced viscoelastic behavior of these structures. The characteristic stress-relaxation response observed in all investigated meshes, comprising an initial rapid stress decay followed by a slower relaxation phase, is also consistent with previous reports [34,40]. The weighted average relaxation times obtained in the present study (416–568 s) indicate that the dominant viscoelastic response occurs within the first several minutes after loading, which is in agreement with previous observations of the time-dependent behavior of polypropylene mesh structures [34,40]. While earlier studies primarily described relaxation behavior in terms of stress reduction, the present work additionally quantified relaxation kinetics using a Prony-series model, enabling characterization of multiple relaxation processes occurring over distinct time scales.

In contrast to the strong anisotropy observed in mechanical parameters such as tear strength, stiffness, and initial stress, stress relaxation behavior displayed a more uniform pattern. Although the absolute stress values ($\sigma_{0}$ and $\sigma_{r}$) depended significantly on the cutting direction, the normalized parameters—relative stress reduction (R) and normalized residual stress ($\sigma_{r}^{nor}$)—remained largely independent of orientation for most meshes. These findings suggest a partial decoupling between structural anisotropy and time-dependent mechanical behavior: while fiber architecture controls the magnitude of the load-bearing response, the relative stress relaxation kinetics are only weakly affected by mesh orientation.

The Prony series analysis further revealed a multi-stage relaxation process. The short-term relaxation time ($\tau_{1}$) likely reflects rapid local structural adjustments of the mesh architecture, whereas $\tau_{2}$ and $\tau_{3}$ correspond to progressively slower relaxation processes. One of the relaxation processes identified in the multi-exponential model may be associated with the intrinsic viscoelastic response of polypropylene fibers, whereas the remaining components may be related to structural rearrangements within the mesh architecture. Because the present study focused on commercially available surgical meshes rather than isolated fibers, direct verification of these contributions was beyond the scope of the work. Future studies involving separate characterization of polypropylene fibers could help distinguish the relative contributions of fiber-level and structure-level relaxation processes.

The absence of significant directional differences in $\tau_{2}$ and $\tau_{3}$ indicates that the long-term relaxation response is less sensitive to mesh architecture than the immediate mechanical response. This observation is consistent with the comparable weighted average relaxation times obtained for all investigated meshes. The multi-stage relaxation kinetics observed in the present study are consistent with previous investigations of the long-term mechanical response of surgical meshes [34]. Similar observations have been reported for other polymer-based biomaterials, where viscoelastic behavior shows lower sensitivity to structural orientation than mechanical stiffness and strength [40].

From a clinical perspective, the observed anisotropy may have important implications for mesh implantation. The results indicate that the mechanical performance of some surgical meshes is highly sensitive to orientation, particularly in the case of Yavo Soft and Yavo Normal. Since commercially available meshes are typically not marked to indicate their principal material directions, surgeons often rely on manual assessment of stiffness, personal experience, or intraoperative handling characteristics when positioning the implant. While such an approach may be sufficient for nearly isotropic meshes such as Optomesh Thinlight and Macropore, it may lead to non-optimal orientation in highly anisotropic materials.

Consequently, implantation orientation may influence the mechanical response of the implant under loading conditions. However, the extent to which these mechanical differences affect clinical outcomes remains unclear and requires further investigation. Although biomechanical studies have highlighted the importance of mesh orientation, standardized clinical recommendations regarding the implantation direction of anisotropic meshes remain limited. Therefore, the present findings suggest that mesh orientation may represent a factor worthy of consideration during implantation, particularly for highly anisotropic meshes. Future studies combining biomechanical testing with biological and clinical evaluation are needed to determine the clinical significance of mesh anisotropy and implantation orientation.

Beyond the influence of mesh architecture and orientation, broader methodological challenges also complicate the interpretation and comparison of biomechanical data. The lack of standardized testing methods remains a significant limitation in the field. Differences in testing conditions, strain levels, specimen geometry, and specimen orientation complicate direct comparison of results between studies [30,32]. This highlights the need for unified experimental protocols that would enable more reliable evaluation and comparison of mesh performance. Standardization would also facilitate the translation of biomechanical findings into clinically meaningful recommendations.

Another limitation of this study is the method used to calculate stresses, which employs a simplified rectangular cross-section model based on the specimen’s nominal dimensions. Since surgical meshes are highly porous structures, the actual load-bearing area is significantly smaller than the calculated geometric cross-sectional area. As a result, the obtained stress values should be interpreted as apparent (effective) stresses, rather than as the actual stresses occurring within the polypropylene fibers. The approach adopted does not account for local variations in pore geometry or fiber orientation, nor does it account for stress concentrations resulting from the mesh structure. Nevertheless, this method provides a practical and reproducible way to compare the mechanical behavior of different types of meshes under identical test conditions.

From a wider perspective, the limitations identified for conventional polypropylene meshes, including sensitivity to structural design and anisotropic mechanical behavior, have stimulated the development of advanced biomaterials and next-generation surgical meshes with adaptive mechanical properties. Recent concepts include bioactive, nanocomposite, and stimuli-responsive systems capable of adapting to the local mechanical environment, thereby improving tissue integration and long-term functionality [16,24,41]. Nanocomposite biomaterials are of particular interest because they combine favorable mechanical performance with improved biological integration, controlled degradation, and stimulation of tissue regeneration, thereby addressing several limitations associated with conventional polypropylene meshes [42] identified in the present study. At the same time, factors such as surface modification, sterilization procedures, and material aging have been shown to affect the mechanical performance of surgical meshes. These findings are consistent with previous studies demonstrating that mesh architecture and post-processing procedures can substantially influence the mechanical response of polypropylene meshes [38,43]. Therefore, future investigations should integrate structural characterization, time-dependent mechanical behavior, and long-term degradation phenomena to improve prediction of in vivo performance and implant longevity.

Overall, the present study demonstrates that both structural design and viscoelastic behavior must be considered simultaneously when evaluating surgical meshes, particularly in the context of anisotropy and long-term mechanical performance. Mechanical properties were characterized using two complementary approaches. Initial stiffness was determined from the ramp phase of the test, whereas stress relaxation behavior was described using a normalized Prony series. This separation enables independent evaluation of structural stiffness and time-dependent viscoelastic response. The weighted average relaxation time ranged from approximately 400 to 600 s, indicating that the dominant viscoelastic response of the investigated meshes occurs within the first several minutes after loading. This time scale may be relevant for the initial biomechanical adaptation of the mesh following implantation. Materials with shorter relaxation times may better conform to surrounding tissues, whereas those with longer relaxation times may provide mechanical support over a more extended period. Together, these findings demonstrate that optimal mesh performance depends not only on tensile strength and stiffness, but also on the interplay between structural anisotropy and viscoelastic behavior, both of which should be considered during material design, testing, and clinical application.

The present study has several limitations. All experiments were performed under in vitro conditions using commercially available meshes tested at room temperature under controlled laboratory conditions. The adopted methodology does not account for tissue integration, healing processes, inflammatory response, degradation, or other biological factors that may influence in vivo performance. Therefore, the reported results should be interpreted as a comparative mechanical characterization rather than a direct predictor of clinical outcomes.

## 5. Conclusions

The mechanical performance of polypropylene surgical meshes was strongly influenced by structural architecture and loading direction. Significant differences in tear strength, stiffness, and apparent Young’s modulus were observed among the investigated mesh types and loading directions. Yavo meshes exhibited the highest degree of anisotropy, whereas Optomesh Thinlight showed nearly isotropic behavior. In contrast, normalized stress-relaxation parameters and relaxation kinetics were largely independent of orientation, indicating that time-dependent viscoelastic behavior is less sensitive to structural anisotropy than the immediate mechanical response. These findings highlight the importance of considering both anisotropy and viscoelastic behavior for a comprehensive mechanical evaluation of surgical meshes.

## Figures and Tables

**Figure 1:**
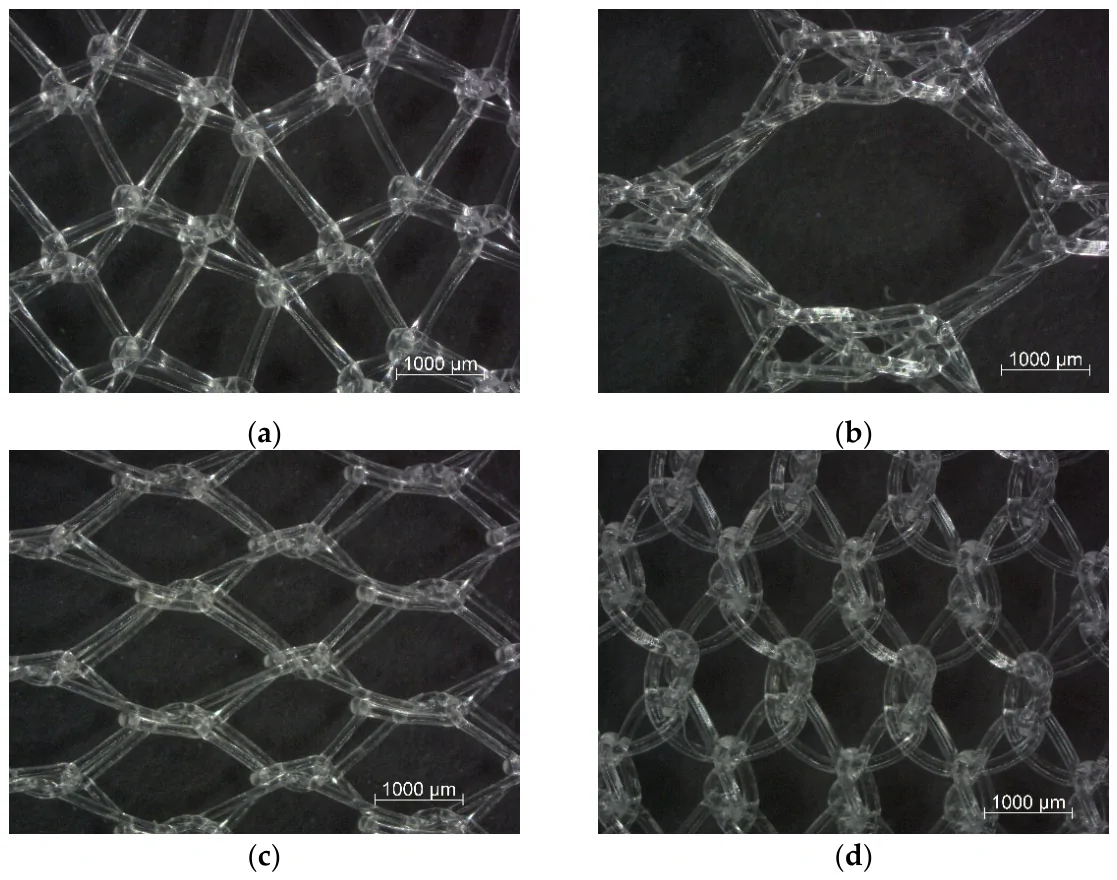
Surgical meshes used in the tests: (**a**) Optomesh Thinlight, (**b**) Optomesh Macropore (Tricomed Sp. z o.o., Łódź, Poland) as well as (**c**) Yavo Soft, (**d**) Yavo Normal (Yavo Sp. z o.o., Bełchatów, Poland). Magnification: 150×.

**Figure 2:**
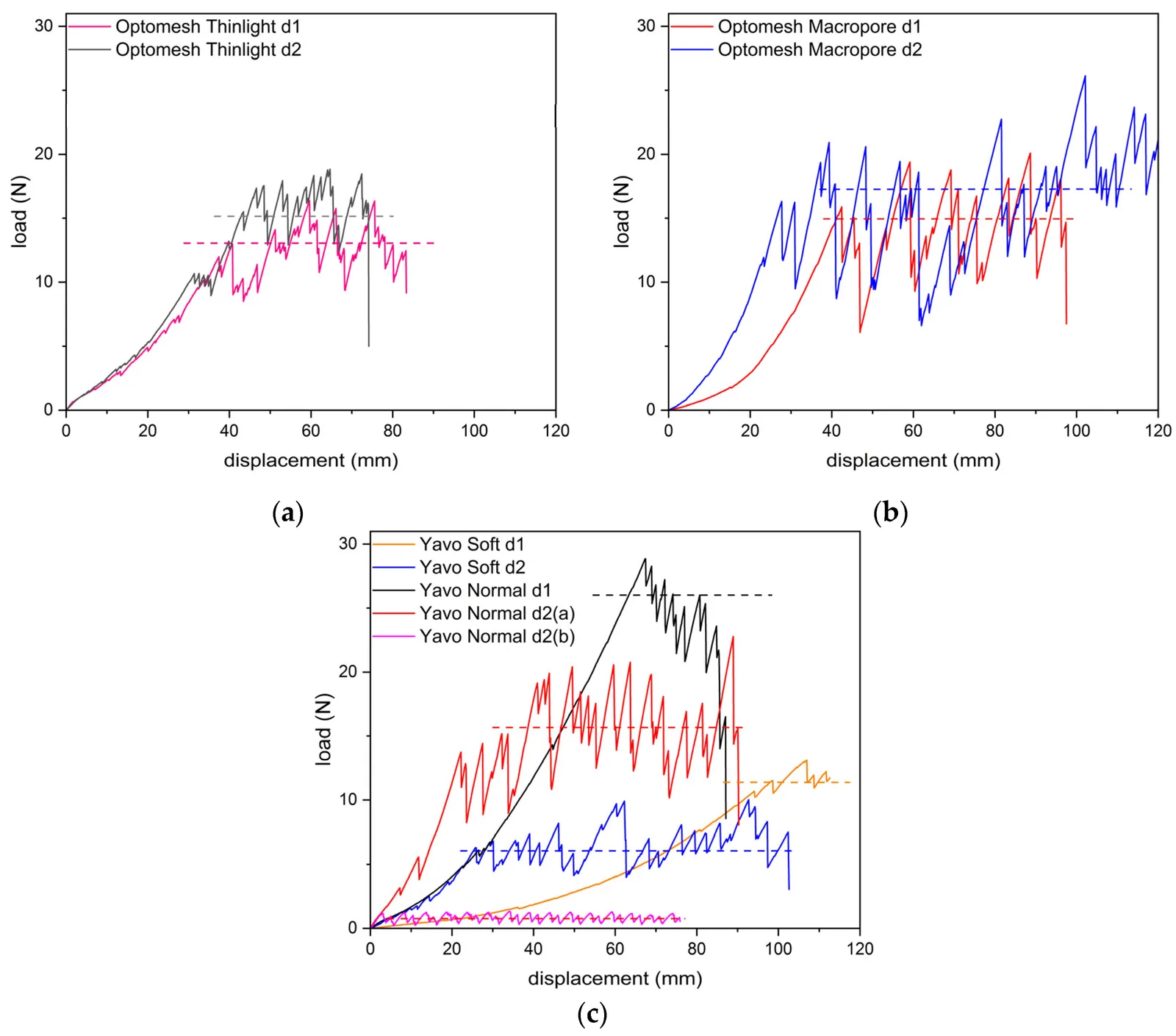
Tear test curves of surgical meshes: (**a**) Optomesh Thinlight, (**b**) Optomesh Macropore, (**c**) Yavo Soft and Yavo Normal ((**a**) specimens without node intersection, (**b**) specimens with node intersection). Note: d1 and d2 denote the two orthogonal loading directions of the surgical mesh specimens. The dashed line indicates the mean values of the tearing force.

**Figure 3:**
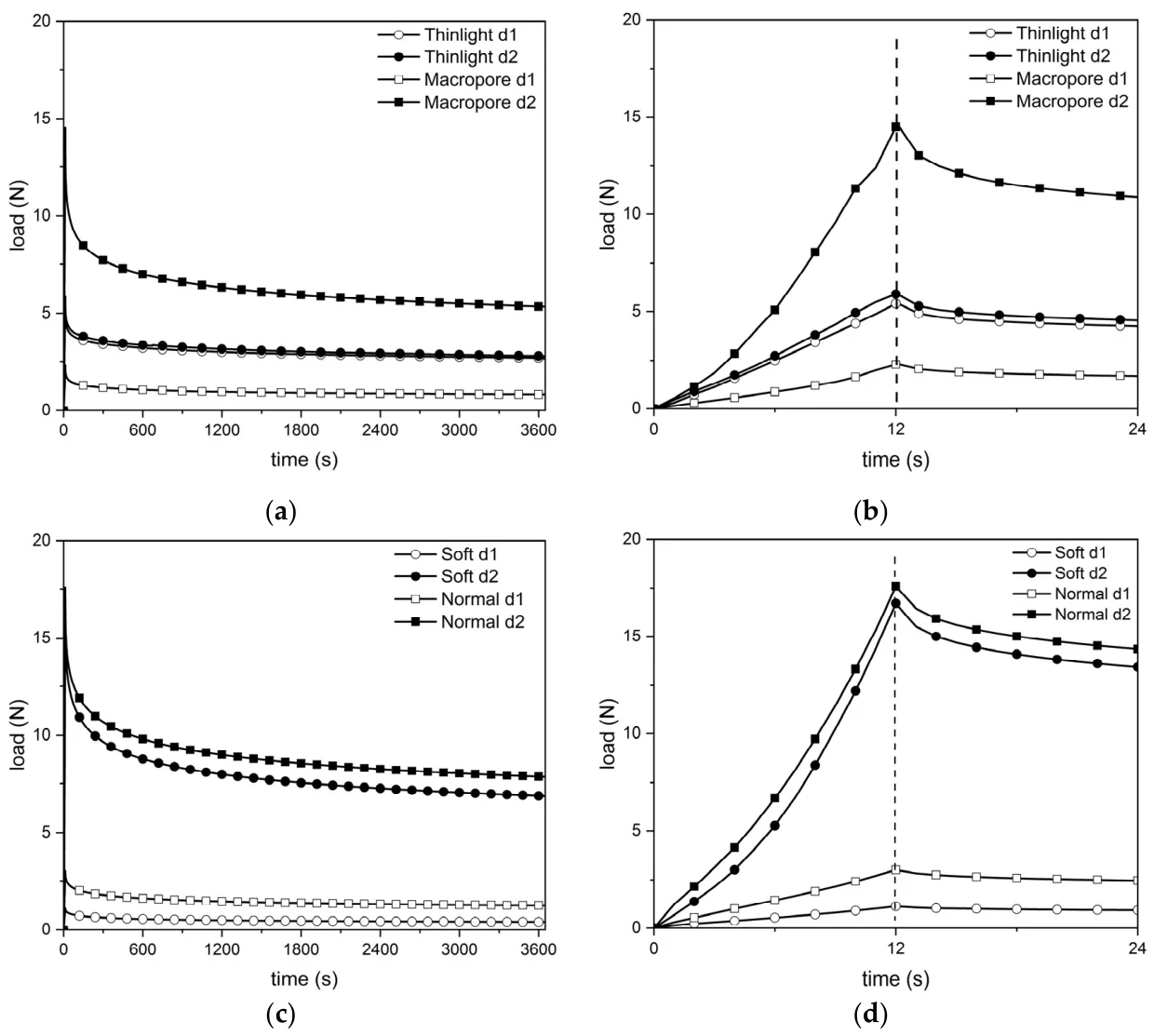
Mean stress relaxation curves: (**a**) Optomesh meshes (Thinlight and Macropore)—full-time range, (**b**) Optomesh meshes (Thinlight and Macropore)—reduced time scale, (**c**) Yavo meshes (Soft and Normal)—full-time range, (**d**) Yavo meshes (Soft and Normal)—reduced time scale. Note: d1 and d2 denote the two orthogonal loading directions of the surgical mesh specimens. The vertical dashed line indicates the transition between the ramp phase and the relaxation phase.

**Figure 4:**
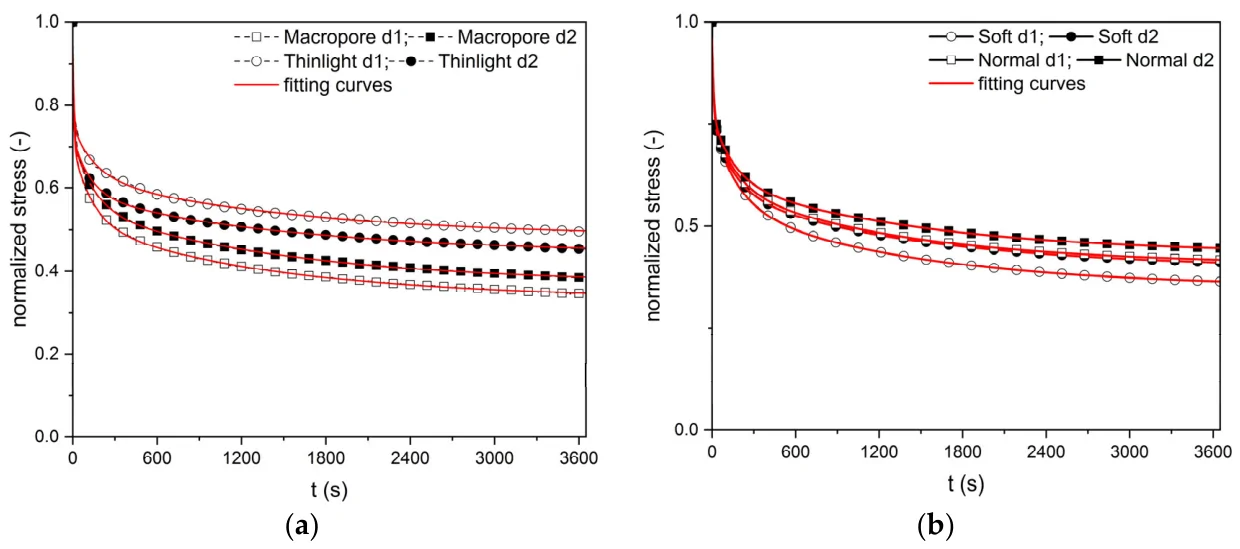
Normalized stress-relaxation curves and corresponding Prony series fit for surgical meshes: (**a**) Optomesh meshes (Thinlight and Macropore) and (**b**) Yavo meshes (Soft and Normal). Note: d1 and d2 denote the two orthogonal loading directions of the surgical mesh specimens.

**Table 1:** Characteristics of surgical meshes used in the study [35,36].

Properties	YAVO Soft	YAVO Normal	Tricomed Optomesh Macropore	Tricomed Optomesh Thinlight
Grammage, g/m^2^	55 ± 10%	85 ± 10%	40	60
Pore size, mm	1.3 × 2.0 ± 10%	1.0 × 1.2 ± 10%	1.5–3.5	1.4 ± 0.1
Fiber diameter, mm	0.15 ± 10%	0.15 ± 10%	0.10–0.15	0.10–0.15
Nominal thickness, mm	0.32 ± 0.01	0.55 ± 0.01	0.61 ± 0.02	0.46 ± 0.01

**Table 2:** Mean values (±SD) of tear strength (TS, N/mm) and degree of anisotropy (α) for surgical meshes as a function of specimen cutting direction.

Surgical Mesh	d1	d2	*α*
Optomesh Thinlight	30.72 ± 3.10	34.77 ± 2.28	0.82 ± 0.09
Optomesh Macropore	28.19 ± 3.07	30.54 ± 5.61	0.94 ± 0.11
Yavo Soft	39.48 ± 0.79	29.35 ± 0.55	1.35 ± 0.02
Yavo Normal (a)	51.82 ± 2.25	31.66 ± 1.27	1.67 ± 0.09
Yavo Normal (b)	-	2.08 ± 0.15	-

Note: (a) specimens in which mesh nodes were not intersected during preparation; (b) specimens in which mesh nodes were intersected during preparation; (-) not determined or not applicable.

**Table 3:** Mechanical parameters determined from the quasi-linear region of the loading ramp (up to 10% strain).

Surgical Mesh	*k* [N/mm]		*E* [MPa]		*α*
	d1	d2	d1	d2	
Thinlight	0.50 ± 0.02 ^a^	0.55 ± 0.05 ^a^	5.40 ± 0.24 ^a^	5.83 ± 0.61 ^a^	0.92 ± 0.10
Macropore	0.17 ± 0.01 ^a^	1.01 ± 0.25 ^b^	1.40 ± 0.14 ^a^	8.38 ± 2.02 ^b^	0.18 ± 0.05
Soft	0.11 ± 0.02 ^a^	0.94 ± 0.02 ^b^	1.75 ± 0.27 ^a^	13.98 ± 1.47 ^b^	0.11 ± 0.01
Normal	0.27 ± 0.04 ^a^	1.30 ± 0.25 ^b^	2.54 ± 0.29 ^a^	11.86 ± 2.25 ^b^	0.21 ± 0.02

Note: Different lowercase letters within the same row indicate statistically significant differences between directions (Welch’s *t*-test, *p* ≤ 0.05).

**Table 4:** Stress relaxation characteristics of surgical meshes (mean values ± SD).

Surgical Mesh	Direction	*σ*_0_ [MPa]	*σ*_r_ [MPa]	$\sigma_{r}^{nor}$ [-]	*R* (Relative Stress Reduction)
Optomesh Thinlight	d1	1.17 ± 0.04	0.58 ± 0.02	0.50 ± 0.00	0.51 ± 0.01
	d2	1.19 ± 0.15	0.60 ± 0.05	0.47 ± 0.00	0.49 ± 0.02
Optomesh Macropore	d1	0.38 ± 0.05	0.13 ± 0.02	0.35 ± 0.01	0.65 ± 0.01
	d2	2.38 ± 0.32	0.87 ± 0.08	0.37 ± 0.02	0.63 ± 0.01
Yavo Soft	d1	0.35 ± 0.05	0.13 ± 0.02	0.39 ± 0.05	0.64 ± 0.02
	d2	5.23 ± 0.85	2.14 ± 0.31	0.41 ± 0.01	0.59 ± 0.01
Yavo Normal	d1	0.52 ± 0.07	0.22 ± 0.02	0.41 ± 0.02	0.58 ± 0.03
	d2	3.14 ± 0.47	1.40 ± 0.22	0.45 ± 0.01	0.55 ± 0.01

**Table 5:** Prony series parameters (Mean ± SD) describing the normalized stress relaxation behavior of surgical meshes in two directions.

Surgical Mesh	Direction	*A*_0_	*A*_1_	*A*_2_	*A*_3_	*τ*_1_	*τ*_2_	*τ*_3_
Optomesh Thinlight	d1	0.45 ± 0.01	0.22 ± 0.01	0.17 ± 0.01	0.13 ± 0.01	6.07 ± 1.12	144.47 ± 50.93	1723.09 ± 183.61
	d2	0.49 ± 0.01	0.22 ± 0.01	0.13 ± 0.01	0.16 ± 0.01	5.93 ± 2.09	137.70 ± 45.79	1483.05 ± 177.96
Optomesh Macropore	d1	0.34 ± 0.01	0.24 ± 0.01	0.18 ± 0.01	0.18 ± 0.01	6.95 ± 1.88	116.94 ± 31.67	1232.70 ± 287.94
	d2	0.36 ± 0.02	0.22 ± 0.01	0.17 ± 0.01	0.19 ± 0.01	7.67 ± 0.33	136.16 ± 7.18	1428.33 ± 42.89
Yavo Soft	d1	0.35 ± 0.02	0.19 ± 0.01	0.20 ± 0.01	0.21 ± 0.01	15.93 ± 1.71	175.37 ± 15.98	1408.1 ± 114.59
	d2	0.40 ± 0.01	0.18 ± 0.01	0.17 ± 0.01	0.19 ± 0.01	12.08 ± 0.65	162.71 ± 6.73	1462.2 ± 46.21
Yavo Normal	d1	0.41 ± 0.01	0.18 ± 0.01	0.17 ± 0.01	0.19 ± 0.01	13.70 ± 2.04	168.6 ± 19.07	1373.08 ± 86.63
	d2	0.43 ± 0.01	0.17 ± 0.01	0.16 ± 0.01	0.18 ± 0.01	11.46 ± 0.27	156.98 ± 4.30	1465.61 ± 26.11

## Data Availability

The original contributions presented in this study are included in this article. Further inquiries can be directed to the corresponding author.

## Abbreviations

The following abbreviations are used in this manuscript:

FDA	Food and Drug Administration
EU	European Union
COD	Coefficient of Determination
SD	Standard Deviation

## References

[1] U.S. Food and Drug Implants and Prosthetics. https://fda.gov/medical-devices/products-and-medical-procedures/implants-and-prosthetics.

[2] European Union (2017). Regulation (EU) 2017/745 of the European Parliament and of the Council of 5 April 2017 on Medical Devices, Amending Directive 2001/83/EC, Regulation (EC) No 178/2002 and Regulation (EC) No 1223/2009 and Repealing Council Directives 90/385/EEC and 93/42/EEC (Text with EEA Relevance).

[3] Köckerling F., Simon T., Hukauf M., Hellinger A., Fortelny R., Reinpold W., Bittner R. (2017). The importance of registries in the postmarketing surveillance of surgical meshes. Ann. Surg..

[4] Antoniadi E., Ferreira N.M., Vaz M.F., Parente M., Ferraz M.P., Silva E. (2025). Innovative strategies in hernia mesh design: Materials, mechanics, and modeling. Materials.

[5] Baylón K., Rodríguez-Camarillo P., Elías-Zúñiga A., Díaz-Elizondo J., Gilkerson R., Lozano K. (2017). Past, present and future of surgical meshes: A review. Membranes.

[6] Klinge U., Klosterhalfen B. (2012). Modified classification of surgical meshes for hernia repair based on the analyses of 1000 explanted meshes. Hernia.

[7] Saha T., Wang X., Padhye R., Houshyar S. (2022). A review of recent developments of polypropylene surgical mesh for hernia repair. OpenNano.

[8] Liu P., Chen N., Jiang J., Wen X. (2019). New surgical meshes with patterned nanofiber mats. RSC Adv..

[9] Özant A., Arslan K. (2023). Synthetic meshes in hernia surgery. Cyprus J. Med. Sci..

[10] Heidari B., Dodda J., El-Khordagui L., Focarete M., Maróti P., Tóth L., Pacilio S., El-Habashy S., Boateng J., Catanzano O. (2024). Emerging materials and technologies for advancing bioresorbable surgical meshes. Acta Biomater..

[11] Mancuso E., Downey C., Doxford-Hook E., Bryant M.G., Culmer P. (2020). The use of polymeric meshes for pelvic organ prolapse: Current concepts, challenges, and future perspectives. J. Biomed. Mater. Res. B Appl. Biomater..

[12] Călinescu B.C., Neacșu A., Martiniuc A.E., Dumitrescu D., Stănică C.D., Roșu G.A., Chivu L.I., Ioan R.G. (2023). Surgical treatments for women with stress urinary incontinence: A systematic review. Life.

[13] Corduas F., Lamprou D., Mancuso E. (2021). Next-generation surgical meshes for drug delivery and tissue engineering applications: Materials, design and emerging manufacturing technologies. Bio-Des. Manuf..

[14] Gao Y., Han X., Chen J., Pan Y., Yang M., Lu L., Yang J., Suo Z., Lu T. (2021). Hydrogel–mesh composite for wound closure. Proc. Natl. Acad. Sci. USA.

[15] Todros S., Pavan P., Natali A. (2017). Synthetic surgical meshes used in abdominal wall surgery: Part I-materials and structural conformation. J. Biomed. Mater. Res. B Appl. Biomater..

[16] Saiding Q., Chen Y., Wang J., Pereira C., Sarmento B., Cui W., Chen X. (2023). Abdominal wall hernia repair: From prosthetic meshes to smart materials. Mater. Today Bio.

[17] Xie Y., Li S., Zhang T., Wang C., Cai X. (2020). Titanium mesh for bone augmentation in oral implantology: Current application and progress. Int. J. Oral Sci..

[18] Antón M., Pérez-González F., Meniz-García C. (2024). Titanium mesh for guided bone regeneration: A systematic review. Br. J. Oral Maxillofac. Surg..

[19] Saha T., Houshyar S., Sarker S., Ghosh S., Dekiwadia C., Padhye R., Wang X. (2019). Surface-functionalized polypropylene surgical mesh for enhanced performance and biocompatibility. ACS Appl. Bio Mater..

[20] Taylor D. (2018). The failure of polypropylene surgical mesh in vivo. J. Mech. Behav. Biomed. Mater..

[21] Fiedler A. (2019). Biodegradation of surgical polypropylene meshes. FASEB J..

[22] Patrocinio D., Pagador J., Galván V., Duarte M., Sánchez F. (2023). Preliminary results on functionalization of polypropylene surgical meshes. Br. J. Surg..

[23] Fontana-Escartín A., Hauadi K., Lanzalaco S., Pérez-Madrigal M., Armelin E., Turon P., Aléman C. (2023). Preparation and characterization of functionalized surgical meshes for early detection of bacterial infections. ACS Biomater. Sci. Eng..

[24] Lanzalaco S., Turon P., Weis C., Mata C., Planas E., Alemán C., Armelin E. (2020). Toward the new generation of surgical meshes with 4D response: Soft, dynamic, and adaptable. Adv. Funct. Mater..

[25] Polyák P., Vadász K., Tátraaljai D., Puskas J. (2024). Preparation of surgical meshes using self-regulating technology based on reaction-diffusion processes. Med. Biol. Eng. Comput..

[26] Farmer Z., Domínguez-Robles J., Mancinelli C., Larrañeta E., Lamprou D. (2020). Urogynecological surgical mesh implants: New trends in materials, manufacturing and therapeutic approaches. Int. J. Pharm..

[27] Young E., Lawson J., Karatassas A., Hensman C. (2025). To infinity and beyond: The promise of data-driven 3D printing of hernia mesh—A primer for surgeons. Hernia.

[28] Olmos-Juste R., Olza S., Gabilondo N., Eceiza A. (2022). Tailor-made 3D printed meshes of alginate-waterborne polyurethane as suitable implants for hernia repair. Macromol. Biosci..

[29] Chacon V., Caballero P., Carrasco P., León D., Margallo S. (2023). Effective porosity of 3D printed surgical meshes. Br. J. Surg..

[30] Civilini V., Giacalone V., Audenino A., Terzini M. (2023). A reliable and replicable test protocol for the mechanical evaluation of synthetic meshes. J. Mech. Behav. Biomed. Mater..

[31] Ibrahim M., Poveromo L., Glisson R., Cornejo A., Farjat A., Gall K., Levinson H. (2018). Modifying hernia mesh design to improve device mechanical performance and promote tension-free repair. J. Biomech..

[32] Weis C., Lecuivre J., Batke B. (2022). OC-035 test methods for surgical meshes—An industry point of view. Br. J. Surg..

[33] Ruiz-Zapata A.M., Feola A.J., Heesakkers J., de Graaf P., Blaganje M., Sievert K.D. (2018). Biomechanical properties of the pelvic floor and its relation to pelvic floor disorders. Eur. Urol. Suppl..

[34] Pashkouleva D., Kirilova-Doneva M. (2024). Solid mechanics experimental investigation of long-term mechanical response of surgical meshes. J. Theor. Appl. Mech..

[35] Yavo Polypropylene Surgical Mesh. https://yavo.com.pl/en/offer/surgical-mesh/non-absorbable-meshes/.

[36] Tricomed Optomesh^®^. https://tricomed.pl/products/optomesh/#show.

[37] Maurer M.M., Röhrnbauer B., Feola A., Deprest J., Mazza E. (2015). Prosthetic meshes for repair of hernia and pelvic organ prolapse: Comparison of biomechanical properties. Materials.

[38] Farr N.T.H., Roman S., Schäfer J., Quade A., Lester D., Hearnden V., MacNeil S., Rodenburg C. (2021). A novel characterisation approach to reveal the mechano–chemical effects of oxidation and dynamic distension on polypropylene surgical mesh. RSC Adv..

[39] Mirjavan M., Asayesh A., Jeddi A. (2017). The effect of fabric structure on the mechanical properties of warp knitted surgical mesh for hernia repair. J. Mech. Behav. Biomed. Mater..

[40] Kirilova-Doneva M., Pashkouleva D. (2019). Long-term mechanical compatibility of polypropylene surgical meshes. J. Mech. Med. Biol..

[41] Andronic O., Palcau A.C., Bolocan A., Dinulescu A., Ion D., Paduraru D.N. (2025). Nanocomposite biomaterials for tissue-engineered hernia repair: A review of recent advances. Biomolecules.

[42] Kirilova-Doneva M., Pashkouleva D., Sopotensky S., Petrova G. (2020). Comparison of pre- and post-implantation mechanical behavior of composite hernia meshes. J. Theor. Appl. Mech..

[43] Lanzalaco S., Weis C., Traeger K.A., Turon P., Alemán C., Armelin E. (2023). Mechanical properties of smart polypropylene meshes: Effects of mesh architecture, plasma treatment, thermosensitive coating, and sterilization process. ACS Biomater. Sci. Eng..

